# A review of the effects of artificial light at night in urban areas on the ecosystem level and the remedial measures

**DOI:** 10.3389/fpubh.2022.969945

**Published:** 2022-09-30

**Authors:** Justine Mushobozi Katabaro, Yonghong Yan, Tao Hu, Quan Yu, Xiang Cheng

**Affiliations:** ^1^Faculty of Architecture and Urban Planning, Chongqing University, Chongqing, China; ^2^Key Laboratory of the Ministry of Education of Mountainous City and Towns Construction and New Technology, Chongqing University, Chongqing, China

**Keywords:** nightscape, artificial light at night, urban areas, light pollution, ecosystem

## Abstract

This paper attempts to realize the balance between humans and ecology in designing the nighttime light environment of urban parks by clarifying the influence of nighttime artificial light on the ecosystem of urban parks. Firstly, we reviewed the effects of nighttime artificial light on individual predation and reproduction of animals and personal growth and reproduction of plants. Secondly, we discuss the impact of individual changes caused by artificial lighting on ecosystem function at the ecosystem and analyze its advantages and disadvantages. The results showed that nighttime artificial light had a double-sided impact on the ecosystem, which would hurt the ecosystem function, but had a positive effect on the green space, which lacked natural light and had high plant density. This paper focuses on the areas with increased application of artificial lighting and rich species of animals and plants in night cities, such as urban forest parks and urban green spaces. It discusses how to reduce the intrusion of artificial lighting on ecosystems and how to make better use of the positive effect of artificial light.

## Introduction

Artificial light has been used for many years to illuminate the nocturnal environment. However, for the past decades, artificial light at night (ALAN) in urban areas has increased drastically with urbanization and socio-economic development (1–3). According to the 2018 revision of World urbanization prospects, the United Nations Department of Economics and Social Affairs (UNDESA) estimated that over 55% of the world population resides in urban neighborhoods. Up to the year 2050, the number of urban dwellers will be rising to 68% (4). Therefore, more spaces are expected to be electrically lit shortly. In 2016, Falchi et al. (5) quantified the magnitude of the world's artificial sky luminance, and their report indicated that ~80% of the world's population is exposed to light-polluted skies. At the same time, they also pointed out that light pollution in Asian countries is growing exponentially, with most of the urban population living in neighborhoods with extreme night brightened- sky and poor night visibility.

ALAN has become a ubiquitous feature of urban settings and is often necessary for human safety, recreation, and other social and economic activities (6). Its wide application in urban environments includes street and traffic lighting for security facilitation, building façade accent lighting for security and aesthetics enhancement, and indoor lighting for extended night activities and shift works. It is also applicable for nightscape and artistic expression, illumination of urban parks and the associated landscape amenities, vehicle headlights, billboards illumination, and other social and commercial activities (7–9).

**Figure 1:**
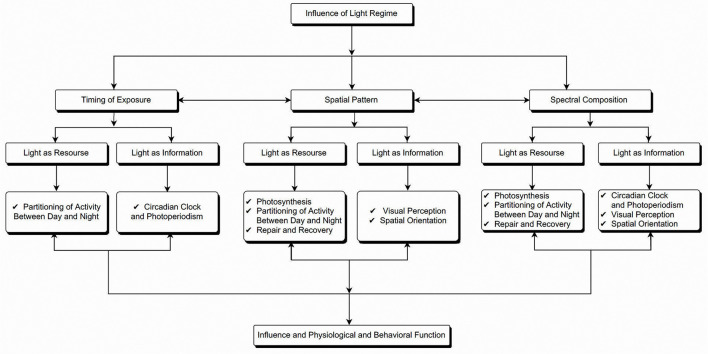
Schematic representation of the influence of light as both resource and source of information in organisms and how it influences their physiological and behavioral functioning.

Although the positive impacts of ALAN on humans are undeniable, however, bright lights at night have come with a price to pay (10). The seasonal variation in day length and stable regime of day and night across 24 h periods has a fundamental role in the physiology and ecology of animals and plants alike (11). The light and darkness cycle act as an energy resource and an information source (Figure 1). Light is the essential resource for the photochemical process in plants, well-known as photosynthesis. On the other hand, light alerts the plant about different weather changes (11–13). The periodic changes, intensity, and spectral composition of natural light provide signals for regulating circadian rhythms of both animals and plants, regulating plants' seasonal phenology and the expression of phenotypic variation, including growth, form, and resource allocation (11, 13). Also, day and night variation is important in temporal niche partitioning among species, spatial orientation, communication, primary productivity, and repair and recovery (14).

Animals, plants, and other organisms depend on each other in their respective environments under a structured system known as the ecosystem (15). The ecosystem consists of a community of animals and plants interacting within their physical environment (16). The components of the ecosystems include both physical and chemical matters, such as soils, water, and nutrients that support the surrounding organisms. These organisms may vary from large animals and plants to microscopic bacteria coexisting in a particular habitat through interaction or mutual dependency (symbiosis). The exchange of both living things and non-living things within the ecosystem is expressed in many ways. These include converting sunlight to energy through photosynthesis, decomposing matters, competition, cooperation, and predation, thus, creating a condition whereby each species is involved in the nutrients cycle and energy flow. Therefore, significant alterations to the ecosystem, like artificial light pollution at night, can initiate damage and imbalances in the entire ecosystem.

Moreover, over the past century, the pervasive use of ALAN connected to human social and economic activities such as industries, settlements, and transportation facilities has altered this natural daily and seasonal cycle of light across the globe (11). It thus affects the well-being of the ecosystem key players. The ever-decreasing natural landscape and wildlife resources of urban areas are progressively causing the instability of its ecosystem (1). At present, the urban artificial light both functional lighting (such as roads, bridges, etc.) and landscape lighting (such as squares, urban park landscape lighting) has produced serious nighttime light pollution, and the more the cities develop and expand, the more severe the nighttime light pollution becomes (2). The impact of nighttime light pollution on ecosystems has long been an urgent problem for the healthy development of cities. In recent years, the increase in artificial light at night has exacerbated the severity and urgency of this problem (3). However, in recent years, studies have shown that artificial light at night does not only have a negative impact on plant growth, but also it has a positive effect. For example, in some urban areas where land is scarce, plants are usually planted at high density, and the buildings' obstructions of natural light often lead to poor plant growth (4). In this situation, some Artificial Lighting measures can be used as a light supplement for better plant growth (6). Therefore, this study attempts to analyze the effects of artificial light at night on the ecosystem by exploring different classes mediated via different causal pathways and extending the debate to the methods which can be adopted to reduce the impact of artificial light on the urban ecosystem while balancing between urban development and ecological stability.

## Materials and methods

In attempting to develop this study, a wide range of most recent literature from different databases such as Web of Science (WOS), PubMed, Engineering Village (EI Compendex), IEEE/IET Electronic Library (IEL), CNKI were reviewed. The review work, analysis, and technical coordination were mainly based on the broad context of biological interdependence in the ecosystems. Moreover, the entire task of literature identification, collection, and review process was implemented according to the following protocols.

### Literature search strategy

First, three research questions were developed to guide the literature search and prioritization process. The formulated research question included;

1. *How Does Artificial Lighting at Night Affect the Well-Being of an Individual Organism? And how Does This Affect the Population, Community, and Ecosystem Health?*
2. *What Are the Characteristics of Artificial Light at Night That Affect Organisms?*
3. *What Are the Alternative ALAN Design Strategies and Policies That Could be Politically, Economically, Socially, and Culturally Viable to Safeguard the Urban Ecosystem?*

The base for literature search keywords was the combination of two main phrases identified from the developed research questions. The initial search was conducted from January to October 2021, and the updated search was conducted from June to August 2022, with a limit of current peer-reviewed articles published in English and Chinese. Access to the database was made through the Chongqing University Online Library (http://lib.cqu.edu.cn/). The keywords for literature search include: “artificial light at night”, “light pollution”, “ecosystem”, “urban area”, “remedial measures”. The search terms are shown in Table 1. These components are required for potentially eligible literature for this search. In addition, a complementary search was conducted to identify articles addressing mechanisms that could be used to minimize the potential impact of ALAN on the ecosystem.

**Table 1:** The search terms used in the literature collection from the selected database.

**Key words**	**Artificial light at night (ALAN)**	Ecosystem*	**Cities**	**Remedial measures**
Search key words	Artificial Light at Night OR Artificial Night Lighting* OR Lighting*, Artificial Night OR Night Lighting*, Artificial OR Artificial Night Sky Brightness OR Light Pollution OR Pollution, Light	Ecosystem* OR Ecologic System* OR Ecological System* OR System*, Ecological OR System*, Ecologic OR Biome* OR Niche, Ecological OR Ecological Niche OR Habitat*	Cities OR City OR Urban Area* OR Town* OR Urban Forest Park	Remedial Measures OR Remedy OR Remedies

The star mark indicates that the two words should be combined to get the desired results from the database.

### Literature selection process and criterion

**Figure 2:**
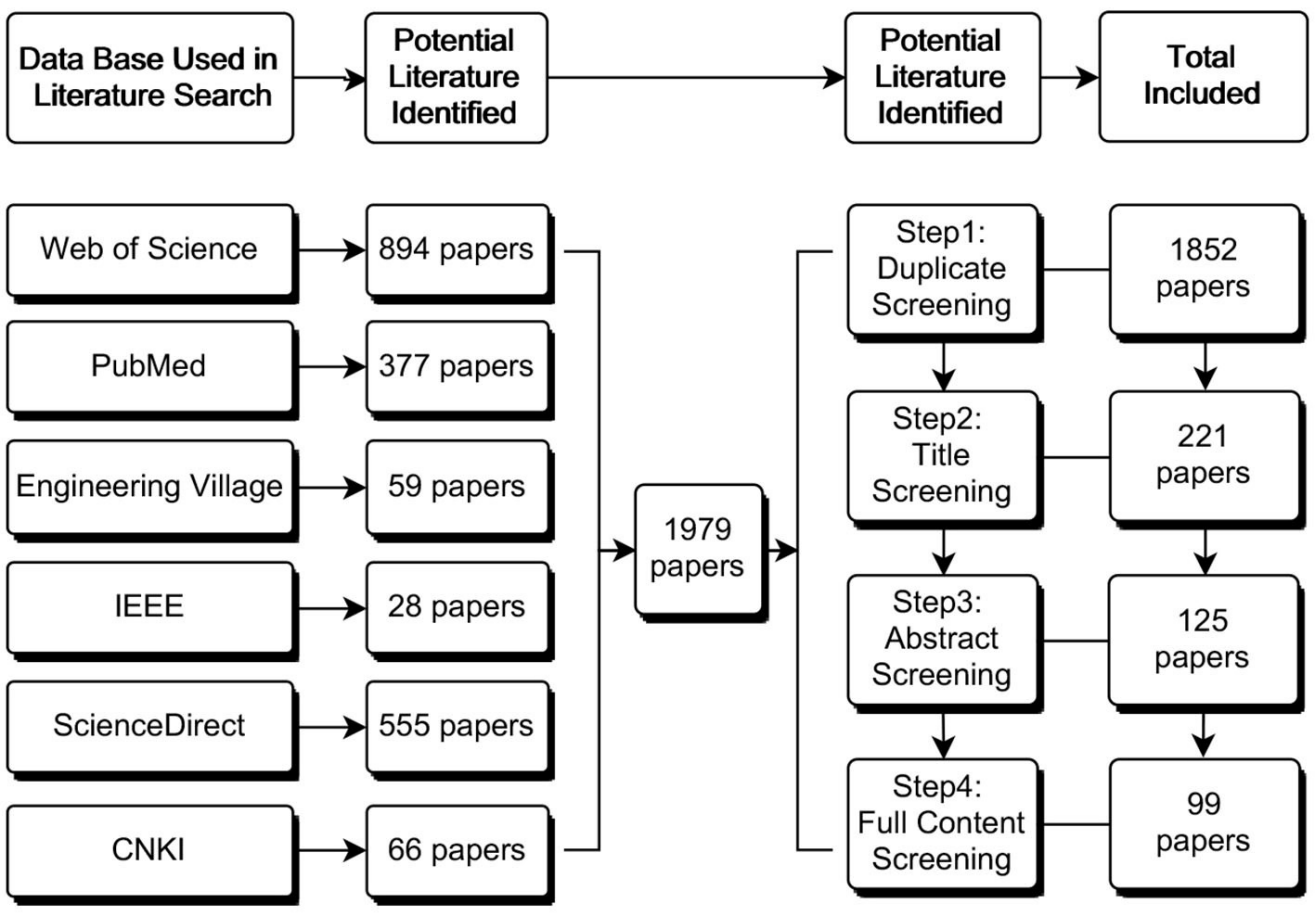
Flowchart of the literature search and selection process.

With the pre-defined search string, we collected 1,979 peer-reviewed articles from six data bases. The research team reviewed the titles and abstracts of all downloaded literature for initial screening. We retained the articles related to the research questions and the study's objective for further review and analysis and excluded those we considered less relevant. The included articles were further filtered based on the relevancy of their contents (see Figure 2). After screening for relevancy and suitability for the present study through a full-text review, we generated 86 pieces of literature that are referred to in this article.

## Results

From the reviewed studies, it was observed that apart from several positive effects of ALAN on some key player groups of the ecosystem, however, its negative impacts on the ecosystem are more significant and can manifest in two primary casual paths. Firstly, ALAN can potentially affect the well-being of an individual organism or species. Secondly, ALAN can alter the community structure and thus modify the ecosystem's function. This section explains how ALAN affects the survival, existence, and well-being of individual organisms and plants and eventually disrupts the entire ecosystem. In addition, because the survival of animals and plants and the steady-state maintenance of the ecosystem are affected by many factors such as climate and geographical conditions, this paper only discusses the impact of artificial light pollution at night on some organisms. In harnessing the critical role played by ALAN on urban social and economic development, the remedial strategies for sustainable ALAN design are reviewed and presented in section four.

### Effect of ALAN on individual animals and plants

#### Influence of ALAN on predatory and foraging behavior of animals

Estimates show that more than 30 % of vertebrates and 60 % of the known invertebrates are nocturnal species (17–19). Nocturnal species highly depend on the light-dark cycle for their visual cues, which are always necessary for navigation (20), protection against predators (21, 22), reproductive success, feeding, and even growth (18). Research shows that nocturnal species are susceptible to light to the extent that even a very weak and temporary ALAN exposure can provoke subtler behavioral responses. ALAN has extensively affected migrating birds, bats, frogs, fireflies, fish, tortoises, etc. (21, 23). The products include the reduced amplitude of migration, poor visibility due to disturbed essentials of navigational signal, disorientation, collision (9, 20, 24, 25), and attraction of new species in a particular environment or repulsion of some species from their habitats. The consequences of these actions affect the foraging behaviors of organisms (26), their communication signals, and reproductive success (27, 28).

Previous research indicates that the illumination intensity, spectral quality of the light, duration, and period of light exposure affect the biochemistry, physiology, and behavior of organisms naturally adapted to stars and moonlight (8, 29). High-intensity urban lighting was found to constrain the association behavior of migrating birds, reducing their flight speeds, altering their flight patterns, and vocalizing frequency (20). Furthermore, bats being the prime predators of nocturnal insects (21), ALAN exposes them to great danger. Bats prefer staying in areas with dark or dim light than bright light. Their preferable nesting sites include caves, abandoned mines, dark rooms, rocky cavities, and big trees. Other nesting sites comprise spaces behind sheltering things and linear landscape features such as hedgerows, riverbanks, canals, and other structures that are protected from light. This light-avoiding behavior is associated with the need to curtail predation risk and poor vision under bright light (21). Therefore, foraging in illuminated areas could expose the nocturnally adapted species such as owls, cats, diurnal hawks, crows, etc., to the risk of predation and sudden mortality due to vehicle smashes (30). Streetlights also put bats and other nocturnal creatures at death risk, especially from being crushed by cars due to their poor vision (21). Illuminating the preferable bats' nesting and foraging sites at the expense of landscaping and tourism has exposed these important creatures to dramatic decreases in numbers.

In another study, Owens and Lewis (31) investigated the effect of ALAN on the predatory behavior of fish and sessile invertebrate prey assemblages. Their report indicated a lower profusion of predatory fish was recorded during the day under natural light. However, under ALAN, their behavior was more predatory than during wild nights. Also, fish were more abundant during natural nights than after applying the electric light. The results can clearly explain the effect that ALAN has on the behavioral response of aquatic creatures. Wild night seashores offer assemblages and protection to most marine ecological communities; however, the presence of ALAN creates day-like foraging opportunities for some diurnal adapted predators and those nocturnal predators who are best favored by relatively brighter conditions.

Coastal beaches are well-known grounds that sea turtles consider safe and suitable for nesting and egg-laying. The emerging hatchlings typically use the night visual signals to locate the bright natural horizon at the sea waters and eventually find their way to the sea. However, as ALAN dominates the coastal habitat of the beaches, the raw visual cues through bright horizons are also lost. This scenario has been reported to significantly disrupt the delicate hatchlings from finding their way to the sea (32). Also, the disorientation of these hatchlings has been associated with an increased mortality rate because of energy loss, dehydration, and predators' visibility (33, 34).

Furthermore, the effect of ALAN on the behavioral response of nocturnal creatures has also been observed in insect species. Insects constitute a large percentage of nocturnal species in the zoological class (35). They are one of the essential organisms in the ecosystem because they play many important ecological allures such as pollination, decomposing matters, and acting as predators and prey (21). Owens et al. reported that the number of insects, especially in urban areas, has significantly declined, thus, posing a perturbing implication for the terrestrial ecosystem (36). Also, reports from other studies have shown that ALAN seriously constrains the wellbeing and fitness of insects, rodents, bats, and amphibians by exposing them to nocturnal predators. To avoid being caught by predators, these species may spend a considerable long time hibernating, reducing the time they need to spend on food searching, shelter location, and other activities (17, 35, 37).

In insect species, the most known effect of ALAN is the flight to light or phototaxis, a phenomenon associated with insects' attraction to the light source's proximity. According to the review report by Baz et al. (35), the eye structure of the insect species allows for sensitive detection of the light intensity, spectral contents, and light directionality. It thus determines the behavior of either positive phototaxy (moving toward the light source) or negative phototaxy (moving away from the light source). Eisenbeis et al. and McGregor et al. indicated that the most consequence of the tendency of the insects' attraction to light sources is massive death, provoked by burns, trapping inside the lamp housing, exhaustion or energy loss exacerbated by overactivity and exposure to predators enticed to the surroundings as a result of the enormous aggregate of insects (9, 38, 39). Liu Yushan investigated the influence of different lighting characteristics on the phototaxis behavior of nocturnal insects in Chongqing urban parks. Whereby it was identified that Diptera, Hymenoptera, Lepidoptera, Homoptera, Coleoptera, and Orthoptera were the main insect species primarily attracted to the light sources (40). Similarly, Perkin et al. investigated the effect of ALAN on aquatic and terrestrial insects along the Spree River of eastern Germany. They identified Diptera as the most affected insect among the collected groups (41).

#### Influence of ALAN on animal reproduction behavior

In most nocturnal species (especially temperate zones), the reproduction process is time-mediated (42). The regular day and night cycle informs most nocturnal ecological players about the appropriate season for synchronizing reproductive activity in their specific habitat (43). ALAN can modify the reproductive physiology of organisms and expose them to great danger and even extinction of some species. The disruption of daily and annual biological and behavioral activities of songbird species, avian bird species, and sea turtles (see Section Influence of ALAN on predatory and foraging behavior of animals) are vivid examples of the negative effect of ALAN on an organism's reproductive phenology community dynamics, and growth. Similarly, previous research has implicated ALAN as one of the toxic exterminators of the firefly species. Fireflies are nocturnal luminous beetles (Coleoptera Lampyridae) that emit different bioluminescence or flashes of light through their particular light-emitting organs in the abdomen, mainly for attracting mates (36). Research indicates that the onset of courtship activity in these species is determined by ambient light intensity. Also, the efficiency of flashing signals is more significant under the dark cloudy night. However, ALAN exposure disrupts the faint light signals that these particular insects produce, hence affecting their media of communication and reproductive success (44–46). Moreover, light pollution disorients and extends the flight periods of the nocturnal luminous beetles, thus exposing them to the habitats predators occupy. Also, the level of natural light, timing, and light sensitivity has always been the predictor of this action in these animal species (47, 48). However, disrupting their natural light cues or signals due to ALAN potentially affects their reproductive success (23). Some studies also suggest that the adverse effect of ALAN on wild birds can go far beyond constraining their reproductive endocrine process (49–52).

#### Influence of ALAN on plant growth and photoperiodism

The regular annual rotation of the earth creates three natural periodic cycles of the light regime. These include the daily cycle of day and night, seasonal variation in day length, and the monthly lunar cycle (10, 16). These cycles provide cues that help organisms anticipate periodic variation in environmental factors such as daily temperature variation and humidity or seasonal variation. The effect of ALAN on the development behavior of plant species has been widely studied. In their research about the ecological impact of ALAN on wild plants, Bennie and colleagues reported that ALAN could affect plants' germination, growth, flowering, phototropism, tissue repair, leaf retention, bud break, and increased disease susceptibility (11). Light, especially sunlight, has always been understood for its importance in enhancing photosynthesis, a natural process always necessary for the growth and nourishment of plant species. However, it has been identified that some other methods, such as flowering, germination, etc., are more sensitive to specific spectral compositions of visible light (8). For example (11, 53, 54) explained the way plants respond to the spectral content of the light by associating the red portion of the light spectrum (625–760 nm) with flowering and the blue amount (360–480 nm) with induced seeds germination. Also, he associated the broad range of the light spectrum (360–760 nm) with photosynthesis or the decomposition of carbonic acid. In addition, photoreceptors in plants typically rely on light to synchronize information regarding the periodic change of season and the time of day (8, 55, 56).

Daylight as an environmental regulator has a continuous full and well-regulated spectral content of blue and red light, which provides a good platform for various natural processes. However, most of the spectrum contents of the ever-increasing artificial lights, such as light-emitting diodes (LED), high-pressure sodium light, etc., negatively affect some plant photoreceptors (35). For example, cryptochromes and Phytochromes are necessary photoreceptors involved in functions such as regulating the circadian clock (35), germination, flowering, growth patterns, proportionality, and blooming regulation, and DNA repair. However, prolonged ALAN exposure to some plant species affects their capacity to synchronize environmental changes and negatively affects their development (8).

Lighting environment characteristics and exposure time are essential in providing these cues in many organisms, including plants. Literature reports that even low-level artificial lighting during the night can disrupt the production and regulation of melatonin hormone in mammals, thereby delaying or advancing the phase shift of the circadian cycle (16). Organisms are either diurnal or nocturnal. Daily organisms are active during the daytime, while nocturnal organisms become dormant and more active at night. Disruption of the light cycle regime entrains the circadian phase, thus, causing numerous biological and ecological consequences in various species. For example, ALAN may induce changes in niche partitioning by prolonging the foraging or predation activity in diurnal species; and suppressing nocturnally adapted species (10, 57, 58). Previous research indicates that tiny nocturnal organisms still exhibit less movement and restricted foraging activity than in the overcast sky, even under the bright moonlight. Therefore, in a particular environment where organisms demonstrate seasonality in their behavior, sustained ALAN exposure significantly reduces their feeding time, diminishes their annual migration amplitude, and subjects them to unfavorable conditions for biological activity (8).

In plants, artificial lighting, particularly those with spectrum contents ranging from red to infrared, tends to prolong the day and alter the flowering patterns. Also, ALAN encourages continued growth, thus preventing some trees from developing dormancy in winter seasons, resulting in poor endurance of the harsh winter conditions (13, 55). Previous research establishes that ALAN exposure on some urban plants can elongate their leaf retention in winter and initiate early commencement of bud burst in the spring, thereby exposing them to the risk of frostiness and pathogens (11, 55). Photoperiodism is the seasonal and light-mediated process that influences leaf shape, surface hairiness (pubescence), and pigment formation in plant species. Also, it affects the defoliation process, root development, dormancy, and the onset of bud burst in temperate plants (55). According to the investigation by Westby and Medley (59), high-intensity light rich in red, blue radiation, and infrared wavelength significantly affects the plants' growth in terms of leaf numbers, color expression, leave morphology, defoliation during winter seasons, and branch biomass. Also, different light sources exhibited other effects on plants. For example, High-pressure sodium lamps had a more significant impact on the plants' leaves' expression than LED lights.

Nevertheless, blue-rich LED lamps significantly affect the leaves' color characteristics and cause burns on the tips of the leaves. Also, the illuminance level of more fabulous than 1100 lx for all types of lamps significantly affected leaves' color expression, life span, and morphology. Similar studies have established that the proximity to ALAN in some agricultural plants such as soya (*Glycine max*) and maize (*Zea mays*) significantly affects their growth and general development. For example, it makes some plants overgrow but fail to flower properly, thus, causing a reduction in crop production (55).

#### Influence of ALAN on plant reproduction

Some temperate adapted species use photoperiod to predict optimal timing of reproduction (42) and plant dormancy development (13, 55). The changes in day length depict a strong biological relevance for their daily and annual rhythms. For instance, the development of the gonads in temperate songbirds is initiated by increasing day length in the early spring, thus meeting an optimal time for reproduction success and survival of both parents and offspring (42, 60). Some studies indicate that sustained exposure of ALAN to these seasonal dependent creatures such as birds tends to advance the onset of gonadal reactivation, thus, causing them to have an extended breeding season (60, 61). According to Phillips et al. (42), extended bleeding seasons expose the offspring to winter's adverse before they can develop strong feathers. Also, extended bleeding may cause them to miss the ideal season for food abundance, reducing the chance for the survival of the offspring.

### Effect of ALAN on ecosystem functioning

An ecosystem is a unified whole composed of organisms and the environment in a particular natural space. In this unified whole, organisms and the environment interact and restrict each other and are in a relatively stable dynamic equilibrium state within a certain period. The functions of an ecosystem include energy flow, material circulation, and information transfer (7). This section discusses the relationship between the impact of ALAN on individual organisms and the changes in ecosystem functions. Since the effects of ALAN on material cycles are relatively indirect and uncertain, this paper focuses on the two primary tasks of ecosystem energy flow and information transfer. The impact will be discussed.

#### Influence of ALAN on ecosystem energy flow

The long-term effect of ALAN on the individual organism to communities also can replicate itself at the ecosystem level *via* alteration of the food web and energy flow. Energy flow in the ecosystem is the primary factor that supports organisms' survival. Mutual dependency between primary producers, consumers, and decomposers ensures a good food chain and food web. However, The energy flow represents how nutrients, organic matters, and prey are transferred across and within ecosystem boundaries (14). The food web structure reflects how species interact, the means of energy flow within the community, and the sustainability of biodiversity within their environment or habitat; thus, a well-functioning ecosystem. The transfer of energy from the primary producer trophic level to the direct consumer creates a natural regulation and balance of the secondary production and biomass accumulation (62). However, ALAN tends to alter how energy is transferred across multiple gradients (14). For example, the delayed leaves fall in deciduous plants could affect the magnitude and timing of nutrients input into aquatic habitats (11, 14). Also, ALAN alters the food chain's length by reducing biodiversity and increasing competition within the habitat.

Furthermore, the glowing night skies make animals adapt to the dark condition to imagine the illumination situation as a full-moon condition, thus remaining hibernated and perhaps underfed. The ALAN condition increases predatory activity intervals in some diurnal species, thus changing the natural prey-predatory relationship (14). While the predators may benefit from the prevailing ALAN condition in the short term, it is not beneficial for the prey that use darkness to hide from predators while foraging. Also, it may be harmful to the predators in the long term because of the altered food web structure (10). In other situations, ALAN attracts more nocturnal creatures to its proximity, leaving different habitats or food patches short of members. The ALAN-induced biodiversity damages and changes in the trophic networks could contribute to the ecosystem functions' degenerations (61, 63).

Maintaining a healthy functioning primary productivity across ecosystems results from well-regulated species interactions. Animals and plants interact naturally, demonstrating an extended mutual benefit across different species and communities. For instance, herbivores depend on plants for their food. Likewise, some animals depend on insects and fruits, while carnivores, fungi, reptiles, and amphibians depend on their fellow animals. Also, plant species largely depend on insects, birds, and decomposers for pollination and nutrient supply. However, the extensive use of ALAN has significantly disrupted the manner organisms interact with each other in their respective habitats. Existing research emphasizes that ALAN can alter the natural environment in which species interact especially predator-prey dynamics, niche partitioning, and competition structure. An expanding knowledge has expounded that predators gain an advantage over prey due to ALAN by exploiting the night niche effectively. Under ALAN, predators multiply their visual foraging success on target attracted to the light sources (64). Also, the bright nights interfere with communities that exhibit resources and niche partitioning across illumination gradients (65).

Several species of frogs are more active when environmental illumination is extremely low. For example, the squirrel's tree frog (*Hyla squirrels*) can perfectly orient and forage in natural conditions and lighting levels lower than 10-5 lux. However, under illumination above 10-3 lux, they stop feeding and remain hibernated to reduce the chance of being detected by visually oriented predators (66). On the other hand, the foraging behavior of the western toad (*Bufo boreas*) is mostly adapted at illumination levels between 10-1 and 10-5 lux, while the tailed frog (*Ascaphus truei*) is best-adapted in the dark part of the night (below 10-5 lux) (67). Although these frog species are not sympatric and may differ in niche dimensions, they demonstrate the foraging behavior and division of illumination gradients. The effect of unexpected lighting conditions on the predator-prey relationship was observed in zooplankton fish predation behavior under dark conditions and the rising moon (Lunar light trap). Predation was significantly higher under moonlight than under darkness (68). Similar observations have been made in artificial lighting conditions. In the study by Bennie et al. (69), the aggregation of harbor seals (Phoca vitulina) and their predation activity on juvenile salmonids were higher under artificial light conditions. They became less when the lights were turned off. These examples illustrate the influence of ALAN on the community structure and the interspecific interactions of organisms in their habitats. The cumulative effect of the disrupted interaction behavior of individual species or communities has potential ramifications on the ecosystem level and its services.

Some research has implicated the behavioral change of individual organisms in response to ambient illumination with the top-down effect of primary productivity in the ecosystem (70, 71). The direct impact on a single species multiplies and manifests itself on other species, eventually affecting the ecosystem's structure and function. For instance, the attraction and congregation of insects on nocturnal lights expose them to sudden mortality leading to a decrease in the population of these principal pollinators (71). Similarly, ALAN attracts insects from other habitats, leaving some areas with reduced insect populations (vacuum effect) (72). In addition, various plant species depend on nocturnal or crepuscular (such as insects, birds, bats, and others) and, to some extent, on night-flying flower visitors for pollination (11). The foraging behavior of these species is initiated and inhibited by specific light levels (73). Thus, reducing insects or changing their regular routines could reduce their frequency of flower visits, thus causing poor pollination and poor productivity. For instance, the foraging activity of the nocturnal carpenter bee (*Xylocopa tranquebarica*) is initiated by nightfall and corresponds with the opening of night-blooming flowers (74). Therefore, ALAN exposure of these bee species could distract their foraging cues and eventually affect this pollination mutualism.

#### Influence of ALAN on ecosystem information transmission

The activities of organisms in the ecosystem are inseparable from the role of information. The stability of the ecosystem is mainly manifested in the regular progress of daily activities of organisms and interspecies relationships. Common information types include physical, chemical, and behavioral information. Increasing evidence demonstrates that ALAN can go far beyond constraining organism behavior, reproductive success, and survivorship to influencing their wellbeing at higher levels of biological organization (75). Research in some nocturnal species indicates that excessive ALAN exposure impedes reproduction success in most organisms and thus, causes population reduction. Other studies emphasize that ALAN has a more permanent effect on organisms' population structure and community composition. Faid et al. (75) studied the influence of street lighting on the design of ground invertebrate communities. They found patches in the streetlights' vicinity and recorded a hierarchy of non-native species. Also, more predators and scavengers gathered around the lighted areas due to the massive population of prey species under and between street lights.

Another study in the marine ecosystem identified that many aquatic insects emerged from the water and flew toward the light source under ALAN conditions. Also, night-active ground-dwelling predators were abundant, and they extended their activity until the daytime (76). Given this, Manfrin and colleagues assert that the changes in the composition of riparian predator and scavenger communities could increase the aquatic and terrestrial species competition; this situation may cascade through the riparian food web and eventually disrupt the marine ecosystem services. Meyer and Sullivan found a similar case in the stream ecosystem. They asserted that the presence of ALAN was responsible for increasing the density and diversity of terrestrial arthropods entering the stream due to reduced emergent aquatic insects and changed community diversity (77). Thus, the changes in the light regime could fatally draw the diurnal species into the night light niche, hence changing the community structure and increasing competition for resources.

Besides, Travis Longcore and Catherine Rich reported a high mortality rate of migratory birds on the elevated and lighted building structures triggered by the lost nighttime visibility due to ALAN's induced sky glow (65, 78). Seasonal migrating or nomadic birds rely on the natural signals of the appropriately scheduled earthly seasons. Thus, the presence of ALAN can cause the migrating birds to start migrating in advance or even very late and miss the perfect climatic conditions for roosting and reproducing (79). It can also cause an individual bird in the migrating groups to become more segregated and face increasing problems finding each other, failing to reach the destination, or some individuals arriving too late at their breeding grounds, thus missing the synchronization with local phenology (17). Also, previous research associates ALAN with advancing the start of the dawn chorus in songbirds. Under natural conditions, the early initiation of the dawn song is regarded as a reliable predictor for the female bird to choose a quality male.

Dark seashore sites and river banks are the preferable nesting areas for turtles and sea birds. However, urban coastal regions have seen unprecedented waterfront development over the past decades. As a result, multiple stressors like artificial structures and many types of night illumination projects such as hotels and recreational areas have been developed (80, 81). Davies and colleagues quantified the extent of light pollution on the marine coastline in some parts of the world (Table 2). Their results vividly indicate that the natural habitats of marine creatures have significantly been modified, and the consequences of this practice have affected the predator-prey interactions in aquatic ecosystems (80).

**Table 2:** The extent of coastal line light pollution around the world.

**Region**	**Kilometers of coastal line affected (Km)**	**Percent of coastal line affected (%)**
Europe	115 383	54.3
Asia (excluding Russia)	113 166	34.2
Africa	18 589	22.1
South America	24 197	15.5
North America	64 356	11.8
Oceania	11 692	7.9
Russia	7,377	6.1
Total	354 760	22.2

Adopted from Nuñez et al. (80). The data was taken in 2010, therefore the figures are subjected to inordinate changes due to the increase in global urbanization and population growth over the past decade. Also, some areas such as Antarctica were excluded from the calculation of the total percentage of the coastal line.

Also, Russo et al. (82) investigated the predatory behavior of fish under electric lighting; their conclusion concurred with Bolton and his colleague that ALAN was responsible for modifying prey-predator interactions with marine creatures. Habitat selection is one of the critical components of the ecosystem. It is the best way to ensure evolutionary fitness in all species. The theories of behavioral ecology indicate that the limits of physiological tolerance and psychological factors such as food diversity and distribution, good prey-predator relationship, conducive breeding grounds, and shelters determine habitat selection. As resources or food patches deplete in one habitat, the organism may start migrating freely in search of new food sources from other food patches in adjacent habitats (36). However, there is increasing evidence that ALAN in urban areas constrains how organisms select their habitats. ALAN has both a deterrent and attraction effect on the movement of organisms across the habitats. Giavi et al. (64) investigated signal habitat choice and activity behavior of crayfish. They found that individuals spent significantly more time hiding in the shelter when subjected to ALAN than when exposed to control conditions. Also, Gavi and colleagues identified that the female seed predators flew away from the plants near the light source to lay eggs on plants either in darkness or exposed to low light intensity (83).

Furthermore, observations show that ALAN causes sudden changes in the movement of avian birds and their habitat selection. Also, studies acknowledge that anthropogenic light influences the stopover of migratory and dispersal birds and alters their habitat selection and settlement at a local and regional spatial scale (84–87). Habitat degradation and non-native species invasion can significantly influence the habitation pattern of the species within a particular community. Also, bats are well known for their immense power of seed dispersal across the tropical forest in the tropical ecosystem. However, ALAN constrains their normal nocturnal activities such as migration and communication. The consequence of interfering with the daily nocturnal activities of these bats has several implications on the tropical ecosystem, such as low rainforest degeneration and loss of biodiversity. Disruption of seasonal navigational cues, population-level changes, or aggregation of individuals in one place may modify the animal interactions such as herbivores and carnivores' interaction, plants and pollinators or seed dispersal interaction, etc. (56, 88). This effect may disturb plant reproduction, ultimately disrupting the food supply chain. For example, over 35% of all crop production depends on pollinators of one kind—usually insects but birds and bats to a lesser extent. However, the entomological literature and other related studies have reported the progressive decline of insect populations, especially in urban areas (36, 89–92). The fall and behavioral change of insects and other organisms translate to the disrupted ecosystem services such as primary productivity and other essentials of the food chain and environmental regulation.

Although studies implicating ALAN's effects on diurnal species' interaction are still very few; however, some of the reports in this area have demonstrated that ALAN can considerably modify the interaction behaviors of diurnal species such as daytime plant-pollinator communities (93). Therefore, we recommend that more research be done to elucidate the influence of ALAN on diurnal pollinator species and other ecological species and communities.

## Measures to reduce the negative effects and enhance the positive effects of of ALAN on the urban ecosystems

Over the past decade, the global awareness of the potential negative consequences of ALAN exposure has grown exponentially. But the increased urban expansion is still exacerbating the cumulative effects of ALAN and a wide range of anthropogenic pressures on wildlife and ecosystems. Although ALAN has a detrimental impact on the ecosystem, it is inevitable in today's urban environment. This is because ALAN facilitates the social and economic activities of the urban residents during the night times. However, it is imperative to adopt some sustainable management options to minimize their impacts on the well-being of the urban ecosystem. While ALAN has detrimental effects on ecosystems, it is unavoidable in today's urban environment. This is because ALAN facilitates city dwellers' social and economic activities at night. However, adopting some sustainable management options is imperative to minimize their impact on urban ecosystems. At the same time, for urban core areas lacking natural light and green space, ALAN can be used as a light supplement to affect the growth of plants in the region positively. How to reduce the negative impact of ALAN on the urban ecosystem as much as possible on the premise of meeting the needs of urban lighting at night and exploring its positive effect on the growth of animals and plants is a topic worthy of follow-up research.

### Clearly identify the impact mechanism of ALAN on the ecosystem

It is essential to widely strengthen public awareness regarding the consequences of poor management of ALAN in the environment. Currently, very few countries and cities have incorporated clauses addressing the issues of ecological impacts of ALAN in the urban lighting planning ordinances, norms, and standards. Most of the lighting design regulations and norms focus on the varying sensitivity of the human eye to various light wavelengths and pedestrians' or drivers' safety, hence, missing the essential knowledge of biological consequences of light to the surrounding organisms, their communities, and the wellbeing of the general ecosystem. Also, the general public is not well informed about the ecological ramifications of light pollution. It is, thus, recommended that the way ALAN is designed and implemented in urban areas should be more regulated by enacting the laws and regulations that address light pollution as a threat to the endocrine of the ecosystem. For example, requirements for lighting project planning consents, development reports, and environmental impact assessment reports should also include clauses for compliance with ecological lighting sustainability (94, 95).

### Optimize the division of urban lighting areas

Over the past decade, the alarming level of light pollution has necessitated the urgent need for safeguarding the ever-decreasing entomofauna, lost navigation cues due to poor visibility of the natural Milky Way, and the disturbing essentials of the ecosystem services. The importance of maintaining the dark sky while counterbalancing energy efficiency, socio-economic benefit, and ecology has attracted the attention of many environmental conservationists. Therefore, in urban night lighting planning, more detailed zoning should be carried out according to the regional characteristics.

In areas where animals and plants are denser, such as urban parks, the lighting area should be subdivided according to the similarities and differences in the sensitivity of animals and plants to light in the area to reduce the negative impact of artificial light at night on the ecosystem. In the planning and design of the night light environment of Zhaomushan Forest Park in Chongqing, Yan Yonghong (8), and others, according to the distribution of ecological resources and visual needs of Zhaomushan Mountain, the entire park is divided into three areas: lighting area, lighting control area and prohibited lighting area. A precise lighting partition, different brightness levels, and lighting methods are used in the area to ensure the lighting demand while minimizing the disturbance of artificial light at night to the ecosystem in the park. Xin and Qingxuan (9) and others put forward the concept of “light ecological function zoning” the goal is to protect the environment by controlling the intensity of outdoor artificial lighting at night in different regions, meet the requirements of the carrying capacity of the ecosystem, and realize the harmonious development of the lighting system and the ecological environment system; The method is to propose the luminous flux limits and corresponding design strategies in each control area according to the different characteristic factors of the ecosystem in the area and the difference in their importance. Ma Jian et al. (10) and others studied and summarized the impact of lighting on the animals and plants in the garden in the night scene lighting design of the Summer Palace. For example, if swifts need to rest in a dark environment, if the illuminance exceeds 10lx, it will produce restlessness, which will increase with the continuous increase of the illuminance; It is not advisable to use light sources with high content of red light and far-red light during the germination and growth period of plants in spring and the maturity, fruiting, and deciduous periods in autumn. The Summer Palace is divided into three lighting levels according to the principle of photobiological protection (i.e., protection of animals and plants) restricted area.

### Applying ecological lighting strategy and light optimization

Although it is known that artificial light at night may have a particularly negative impact on the functions of animals and plants and even the entire ecosystem, for places such as urban parks, it is difficult to avoid the use of artificial lighting at night. For such cases, the lighting strategies and methods can be optimized based on the ecological perspective to achieve the balance between humans and ecology in urban parks' night light environment design. In this regard, there have been some exploratory studies, such as Liu Yushan's research on the impact of artificial light on animals at night. Therefore, using LED light sources with shorter wavelengths is conducive to reducing the effects of artificial light on insects at night; in addition, the installation method of lamps also affects phototaxis. Good selection of plant lighting methods and the use of light-cutting lamps to reduce the spillage of lighting in the space. In terms of the influence of artificial light on plants at night, the research of Liu Xiangqian et al. showed that artificial light mainly affects plant growth from light intensity, light duration, and light quality. Trim trees and shrubs significantly impact the overall development, such as sweet-scented osmanthus trees; LED white light should be used, the maximum illumination should not exceed 1,200 lux, and the full-color temperature should not exceed 4,000 K. At the same time, try to avoid using light sources with high blue light energy in the spectrum.

In addition, where security concern is indispensable, some adaptive lighting control gears such as motion or occupancy sensors can be installed to manage the light timing, intensity, and color. Also, these light control gears can turn off the light when there are no users or security alerts and turn it on when the motion is sensed or a user. On the other hand, this technology can be applicable in street and traffic lighting wherever there is less traffic density, especially at midnight. Moreover, the sensor systems in street lighting can be applied to ensure the lights are turned off or dimmed down when there is enough natural light, such as moonlight.

### Applying ecological friendly lighting technology

While rapid urban growth, population expansion, and industrial development are critical factors for the significant increase in artificial lighting and night sky glow, the technological shift to light luminaries with much higher luminous efficacy is also a problem (90). In recent decades, the desire to reduce energy consumption and fight against global warming has prompted the development of lighting technologies that emit high-intensity light with less electric energy input. For example, the light output of the traditional incandescent lamps is 10–20 lumens/watt, while that of modern low-pressure sodium vapor lamps is 200 lumens/watt. Evidence suggests that plants and animals have varied responses to the spectral power distribution of light. The phytochromes system in plants is more sensitive to wavelengths ranging from 660 and 720 nm. The phototropin and cryptochromes in animals are more sensitive to light spectrum peaks in the ultraviolet (96, 97).

The response of plants and animals to light stimuli differs from human beings. Plants and animals directly respond to the light intensity or the number of photons per wavelength that falls on the photoreceptors. However, the light fixture and lighting design strategies currently applied in most lighting projects facilitate human activities during the night without paying enough attention to other ecological players (12). The study by Rodriguez and colleagues (98) revealed that lamp technology significantly affected the mortality and fatality rate of the studied nocturnally active bird species. Metal halide lamps recording high mortality rate (47%) than light-emitting diodes (29%) and high-pressure sodium lamps (24%).

Also, the report by Willems et al. (40) concurred with Rodriquez and colleagues by indicating that the metal-halide light source attracted twice as many insects as the LED. Light sources of the exact nature. Moreover, the report shows that light with a long wavelength had a more significant influence on the phototaxis of insects than that of a shorter wavelength. Furthermore, the phototaxis increased with the brightness of the light source. Another similar study by Baz et al. (35, 46, 99) revealed that insects' response to light depends on the intensity and spectral composition of the light source. Their study found that many moths were attracted to the light source that emitted light with a short wavelength. Hence, applying the lighting technologies that emit a narrow spectrum of light is likely to have fewer ecological effects. However, specific species, such as nocturnally migratory birds, should be paid attention to because visible lights entice them with long-wavelength radiation. Also, we recommend that a research-based approach could be more significant in lighting design projects. The process could help identify the specific lighting technology that can be applied in a particular environment to safeguard the interests of both human beings and other local ecological players.

Furthermore, lighting mitigation measures and design techniques determine how light affects organisms. For example, reports show that the frequency of collisions between migratory birds and elevated structures such as communication towers and illuminated skyscrapers can be minimized significantly by using flashing lighting than non-flashing ones (94). This effect substantiates that dimming the external lighting appliances or turning off the lights during off-peak hours is one of the viable methods of cascading the consequences of ALAN on the ecosystem players. Identifying and dimming down or reducing the intensity of lighting in some ecological zones of the city could help control the magnitude of the artificially lit areas and sky glow and limit lighting duration. It could also decrease the light spill in the unintended spaces or not envisioned to be lit for a long time. In this regard, this research proposes the wide adoption and practice of the concept of ecological lighting design in the urban built environment to avoid light pollution and trespasses. The idea of low energy, non-intrusive optical moonlight and star lighting system advocated by Yan and Hu (8) when designing the nightscape lighting in the Zhaomu mountain urban forest park in Chongqing shows a promising future of achieving an ecological lighting design. This particular design idea helped reduce the overall brightness of the mountain ridge, avoid light pollution, reduce energy consumption, increase the life span of lighting systems, and minimize light trespasses. Therefore, we recommend that further research can be done in this area.

## Conclusions and recommendations

Animals and Plants exist in a well-coordinated and regulated natural system of mutual dependency known as the ecosystem. The natural cycle of day and night has always been the principal regulator of the ecosystem and the proper functioning of organisms. All organisms depend on this cycle for communication, reproductive success, forage, and immune systems development. In most nocturnal microorganisms, including some plants, the night's natural darkness has been an essential factor in their sustained existence throughout evolutionary times. More importantly, the light-dark cycle informs nocturnal animals about the right time to forage, hibernate, hunt, migrate, and the suitable time for reproductive activity. However, ALAN, especially in urban areas, has increased to an alarming level over the past decades. Its effect on animals and plants has captured the attention of many scholars. This review paper has attempted to analyze the mechanism under which ALAN in urban areas affects the ecosystem. The reviewed literature revealed that ALAN could have enormous effects on the endocrine of the ecosystem in many ways.

Firstly, every species uses light as both a resource and information to regulate their behavioral response and physiological function. The timing of light exposure, spatial pattern, and multiple properties of light, such as spectral composition, intensity, etc., affect the interaction between individual organism physiology and behavior. The timing of light exposure affects the spatial and temporal niche partitioning between diurnal and nocturnal organisms. Also, it entrains the circadian clock and changes in photoperiod, especially in the temperate adapted organism. Moreover, the spatial pattern of lighting exposure and the spectral composition of the light affects photochemical processes, visual perception, spatial orientation, and activity partitioning between day and night. These effects manifest into lost nocturnal navigation cues among seasonal migratory species and dispersal organisms, exposure to predators and sudden mortality, affected foraging behavior, and reduced feeding time. Moreover, changing lighting regimes influence organisms' reproductive success, habitat choices, communication, orientation cues, and interaction.

Secondly, it has been identified that the aggravated effect of ALAN on the physiology and behavioral function of an individual organism species results in the imbalance of the essential factors of the ecosystem. For instance, the influence of ALAN on reproduction success and survival of organisms replicates into their population structure. Also, the tendency of ALAN attraction of different species to its proximity or its deterrent effect of some species from the food patches could affect the community structure and its diversity, thus affecting the interaction pattern of the organisms.

T Hardly for some areas, such as urban parks and green spaces requiring artificial lighting to ensure the visual needs of human nocturnal activities, it is difficult to avoid using artificial light. However, in cities lacking sunlight, plants grown in high density in this area need artificial light at night as a light supplement to promote growth. Therefore, this paper recommends that dark sky conservation be mandatory in the traffic lighting planning ordinance, nightscape design standards and regulations, and all other types of electric lighting design norms and measures to safeguard the urban ecosystem and the earth's future. Similarly, for particular areas in the city, we can adopt some methods, such as lighting zoning and planning, and using eco-friendly lighting strategies and technologies, to balance human needs and ecological protection.

Lastly, we recommend more field research to establish the extent of ALAN's effect on the well-being of different diurnal species, their ecological communities, and the wide range of replication to the ecosystem. Also, more interdisciplinary research should be done to identify the lighting technologies and design concepts that support ecological, social, and economic sustainability.

## Data availability statement

The original contributions presented in the study are included in the article/supplementary material, further inquiries can be directed to the corresponding author.

## Author contributions

JK finished review summary preparation, research methodology, and manuscript writing review and editing. YY contributed to topic conceptualization, manuscript writing review and editing, supervision, project administration, and funding acquisition. JK, TH, and QY involved in the literature search. TH finished review summary preparation, research methodology, drawing figures, and editing tables. QY and XC wrote sections of review summary preparation and research methodology. All authors contributed to manuscript revision, read, and approved the submitted version.

## Funding

This research was supported by the Program for Innovation Team Building at Institutions of Higher Education in Chongqing (Grant No. CXTDX201601005).

## Conflict of interest

The authors declare that the research was conducted in the absence of any commercial or financial relationships that could be construed as a potential conflict of interest.

## Publisher's note

All claims expressed in this article are solely those of the authors and do not necessarily represent those of their affiliated organizations, or those of the publisher, the editors and the reviewers. Any product that may be evaluated in this article, or claim that may be made by its manufacturer, is not guaranteed or endorsed by the publisher.
